# Spatial Autocorrelation of COVID-19 in Slovakia

**DOI:** 10.3390/tropicalmed8060298

**Published:** 2023-05-30

**Authors:** Katarína Vilinová, Lucia Petrikovičová

**Affiliations:** Department of Geography, Geoinformatics and Regional Development, Faculty of Natural Sciences and Informatics, Constantine the Philosopher University, 949 01 Nitra, Slovakia; lpetrikovicova@ukf.sk

**Keywords:** Slovakia, district, COVID-19, spatial autocorrelation

## Abstract

The pandemic situation of COVID-19, which affected almost the entire civilized world with its consequences, offered a unique opportunity for analysis of geographical space. In a relatively short period of time, the COVID-19 pandemic became a truly global event with consequences affecting all areas of life. Circumstances with COVID-19, which affected the territory of Slovakia and its regions, represent a sufficient premise for analysis three years after the registration of the first case in Slovakia. The study presents the results of a detailed spatiotemporal analysis of the course of registered cases of COVID-19 in six periods in Slovakia. The aim of the paper was to analyze the development of the number of people infected with the disease COVID-19 in Slovakia. At the level of the districts of Slovakia, using spatial autocorrelation, we identified spatial differences in the disease of COVID-19. Moran’s global autocorrelation index and Moran’s local index were used in the synthesis of knowledge. Spatial analysis of data on the number of infected in the form of spatial autocorrelation analysis was used as a practical sustainable approach to localizing statistically significant areas with high and low positivity. This manifested itself in the monitored area mainly in the form of positive spatial autocorrelation. The selection of data and methods used in this study together with the achieved and presented results can serve as a suitable tool to support decisions in further measures for the future.

## 1. Introduction

The COVID-19 pandemic is unprecedented in modern world history and has triggered a flurry of research activity across the health-related sciences. This effort has produced an equally unprecedented flood of spatially referenced epidemiological data [1]. Since 2019, humanity has been threatened by the severe acute respiratory syndrome virus (SARS-CoV-2) pandemic, which causes COVID-19. Since the first case, which was registered in Wuhan, China, in December 2019, the global spatial dynamics of the disease infection are changing. The disease moved very quickly from Asia to the West to Europe and the United States, South America, and finally to the whole world [2]. As of 2 April 2023, there was a total of 685,887,601 cases and 6,844,035 deaths registered in the databases worldwide (https://www.worldometers.info/coronavirus/) accessed on 10 April 2020. COVID-19 is a highly pathogenic and transmissible viral infection that, according to current research, first appeared in the Chinese city of Wuhan [3]. Coronaviruses are a large group of pathogens, and most of them cause mild respiratory infections. An example is a cold. Coronaviruses can also be deadly. They include the coronavirus (severe acute respiratory syndrome) and COVID-19 (coronavirus disease 2019) [4]. After the outbreak of the COVID-19 pandemic, the scientific field remained active and has paid attention to this epidemic, not only at its beginning but also three years after the first case was recorded. The rate of infectious diseases in the world is on the rise, which can be considered a consequence of poor hygiene measures in terms of environmental conditions. It is important to know the level of understanding of infectious diseases, which are a reflection of the geographical area. Therefore, it is very important to pay attention to the different factors related to health in different regions of the world [5]. COVID-19 has become a new subject of interest in several scientific disciplines. Analyzing it can be complicated to a certain extent, but despite this fact, the more and more professional public is devoted to researching this issue from various points of view [6]. According to Rose-Redwood et al. [7], the COVID-19 pandemic has a completely spatial nature, with value given to geographical theory and practice. All this happens under the assumption of critical (what is happening) and normative (what should happen). This is complemented by applying results (making things happen). The importance of spatial perspective is very useful for understanding the nature of the COVID-19 disease. It is documented for us by studies that are published in various regions of the world. As an example, we present studies from Italy [8,9,10], Turkey [11], Algeria [12], India [13], Indonesia [14], Kuwait [15], China [16], and Canada [17]. In the space of the V4 countries, contributions were made from the environment of the Czech Republic [18,19,20], Poland [21], Hungary [22,23], and Slovakia [24,25,26].

Slovakia is a country with large regional differences, which result from historical, geographical, and cultural differences, but mainly from different levels of economic development. An important role is played by the composition of the population, which gives rise to differences in educational level, social area, and overall economic performance of the regions. To a large extent, it also concerns population development and its individual demographic processes and structures. It turns out that the ongoing transformation of family and reproductive behavior has deepened many of them. Since the beginning of the new millennium, we have witnessed the halt or termination of some established development trends. In some cases, the opposite tendencies that the 1990s brought with them are even being enforced. It seems that the main and most dynamically ongoing part of the transformation of family and reproductive behavior is in the past, and the new model of demographic reproduction is stabilizing. In addition, we are witnessing the extension of life and an increase in the role of migration in population development, which, together with irregularities in the age structure, is reflected in the dynamism of population aging. As for the development of Slovakia’s urbanization, it has changed significantly in the last decade. It manifests itself in the form of concentrated deconcentration and with certain suburbanization tendencies when part of the population from large cities relocates to satellites in their catchment areas [27].

One of the important aspects of the spread of diseases is predicting possible scenarios, which Johnson et al. [28] presented using the example of the European Union. According to the authors, it was important for countries to step up their risk communication efforts and review their pandemic preparedness plans. The spatiotemporal aspect of the COVID-19 pandemic from the regional view of Europe and its countries was analyzed by Hass and Arsanjani [29]. Given the global, geographic spread of the virus and its local spread in many countries, as well as the nature of virus transmission, it is important to understand the spatial mechanisms of spread. This depends on several factors such as distance, demographic, and social characteristics of the infected area. The spatial analysis provides a better understanding of infection transmission routes. Consequently, it enables decision-makers to design and implement effective health and mitigation measures to reduce the risks associated with the pandemic [30].

As reported by Fatima, M. et al. [31], at the beginning of the pandemic outbreak, most of the published studies were conducted in Asia and America. Currently, there are a sufficient number of comparable spatial studies with geographically detailed data in other areas of the world.

One of the possibilities for analyzing the issue of COVID-19 is the use of spatial autocorrelation techniques. According to Haider et al. [32], geographic information science (GIS) has proven to be a unique tool that has extremely valuable insights into a variety of research, including the monitoring of the COVID-19 disease. The importance lies in analyzing the spatiotemporal aspect. The geographic distribution of the epidemic, which can be analyzed using GIS and spatial statistics, is considered an important characteristic. Disease dynamics provides geographic information about an outbreak and can provide insight into disease trends and outbreaks. At the same time, it provides ways to further evaluate the associated risk. The geographical analysis of the virus is based on the question “where is the outbreak and how is it spreading”. Epidemic studies use GIS to determine this. At the same time, it is used for a very detailed examination of disease concentrations. This phenomenon was clearly described by spatial autocorrelation, which is widely applicable in the context of the spread of COVID-19 in any nation. In China, previous research on the distribution of COVID-19 was conducted using the spatial autocorrelation technique using the Moran Index test [33]. The findings showed that cases in one part of China were affecting other parts of the country, suggesting that the COVID-19 pandemic was spreading spatially. Another example of the use of descriptive and spatial analyses of quantitative epidemiological data on the example of francophone West African countries (Benin, Burkina Faso, Ivory Coast, Guinea, Mali, Niger, Senegal) is provided by [34]. Identification, location, size, and risk of purely spatial and spatiotemporal clusters for high incidence of tuberculosis in the Gurage Zone of southern Ethiopia is found in [35].

## 2. Materials and Methods

**Figure 1:**
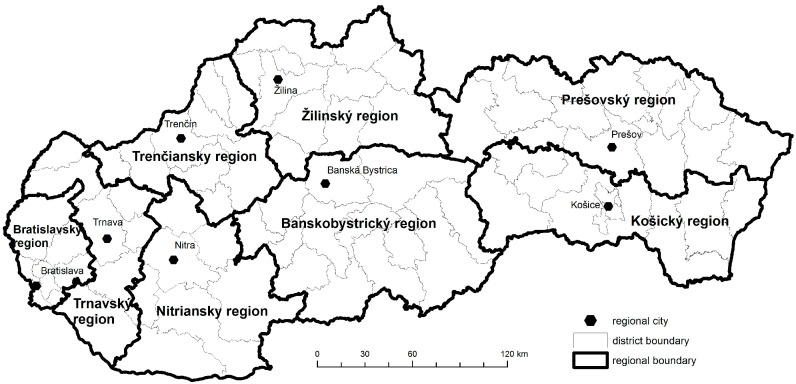
Regional division of Slovakia.

The development of the spatial divergence of the COVID-19 disease at the global level, but also the growth and increase in regional differences in individual countries, represented a rather significant problem. Slovakia could also be included among these countries. The very nature of the available data on the disease of COVID-19, as well as the application of analytical approaches, includes the contribution from a methodological point of view to retrospective analytical cross-sectional studies. These are typical for spatially oriented, epidemiological, and research areas. For the analysis, the number of people infected with COVID-19 was converted to 10,000 residents from the registration of the first case in Slovakia on 6 March 2020 to the present on 6 March 2023. Our observation units for monitoring the spread of the disease with the disease COVID-19 were the districts of Slovakia. The total number of districts in Slovakia is 79. The regional division of Slovakia is represented in Figure 1.

The relevant analytical view was applied separately for 6 time periods. We determined the investigated periods according to the individual waves of the pandemic that were defined in Slovakia. An important role in the analysis of individual periods was played by measures such as closing schools, vaccinations, and others that were gradually introduced during the pandemic. Data on the number of infected people were available from the page of the Institute of Health Analysis-COVID-19-data [36].

First period—between 6 March 2020 and 30 September 2020;

Second period—between 1 October 2020 and 5 March 2021;

Third period—between 6 March 2021 and 30 September 2021;

Fourth period—between 1 October 2021 and 5 March 2022;

Fifth period—between 6 March 2022 and 30 September 2022;

Sixth period—between 1 October 2022 and 6 March 2023.

There are many spatial studies on COVID-19 that use spatial statistics [37,38]. This method is quite often used in professional research. Its history goes back to the 1940s when the presence of positive spatial autocorrelation of cancer mortality in England and Wales was pointed out by Cruickshank [39]. According to Jaber et al. [40], significance estimation is important for showing spatial and transient patterns of the spread of COVID-19. The following is an estimate of its changes over time. Demographic, environmental, and socioeconomic fluctuations play an important role, being able to accelerate the transmission of infection. In this state, GIS is a broad set of spatial statistics that play an important role in planning the spatial and transient patterns of COVID-19. From a policy perspective, this estimate is important to aid policymakers to improve their plans and strategies. The importance of spatial autocorrelation is pointed out by Freitas, W.F. et al. [41]. The authors state that spatially correlated data are geospatial data with spatial autocorrelation and variability that originate from each region and have adjacency to another region. Mortality rates from COVID-19 and their geographical associations with various socio-economic and ecological determinants in Tehran through the use of spatial techniques apply [42]. There is also a cross-sectional study from the Iranian environment that examined spatiotemporal patterns in northeastern Iran from 2016 to 2020. Statistical, spatio-temporal scans and spatial interaction analysis were used in it. The evaluation was carried out using geographic information systems [43].

The exploratory spatial data analysis method was used to verify whether the observed value of a unit has a spatial correlation with the observed values of its neighboring units [44]. The global Moran’s I index is used to measure the global spatial correlation, while the local Moran’s I index in LISA (local indicators of spatial association) was used to measure the local spatial correlation [45].

According to Nazia et al. [17], the global and local Moran’s I tests were run using the first-order Queen’s contiguity spatial weights matrix that uses the values from all first-order neighboring neighborhoods to determine whether the area has a higher or lower mean assessing the degree of spatial autocorrelation.

When there is a grouping of significantly different values, we speak of negative spatial autocorrelation. When similar phenomena or attributes are located closer in space, we speak of positive spatial autocorrelation. When the data are located in space so that nearby values are not in any relationship, the analyzed values are statistically insignificant [46]. In this study, we analyzed the variable using Moran’s index. For Moran’s index:

(1) $$I=\frac{n\left(\sum_{i=1}^{n}\sum_{j=1}^{n}w_{ij}\left(x_{i}-\overset{-}{x}\right)\left(x_{j}-\overset{-}{x}\right)\right)}{\left(\sum_{i=1}^{n}\sum_{j=1}^{n}w_{ij}\right)(\sum_{i=1}^{n}{\left(x_{i}-\overset{-}{x}\right)}^{2})}$$

Moran’s index (*I*) values range from (−1) (perfect variance) to (+1) (absolute correlation). The closer the value of *I* is to 1, the more positive spatial autocorrelation is indicated. The closer the value of *I* is to (−1), the more negative spatial autocorrelation is indicated. Different degrees of spatial autocorrelation can be present within the same ensemble. At the same time, both positive and negative autocorrelation can occur in the same dataset.

Xie Z, et al. [47] developed the mentioned global test of spatial autocorrelation into a series of local indicators called LISA (local indicators of spatial association). It is used to detect local clusters of positive and negative autocorrelation. Within LISA, five different groupings can be identified (high–high, low–low, high–low, low–high, and not significant).

For the analysis of the detection of specific spatial clusters, the local version of Moran’s I criterion was used. It evaluates the level of autocorrelation of a spatial statistical quantity between a given point in space and its surroundings. The relevant indicator is suitable for locating units with relevantly high (i.e., above average)/resp. low (i.e., below average) values (so-called positive spatial autocorrelation). Another case occurs if it is characterized by sudden level breaks in the spatial distribution of the phenomenon (negative spatial autocorrelation). The statistical interference of all three applied indicators (general G-statistics, Moran’s *I* criterion, and local Moran’s *I*) is based on the calculation of the Z-statistics concept [48].

*MoranI:*

(2) $$I=\frac{N}{\sum_{i}^{N}\sum_{j}^{N}w_{i,j}}\frac{\sum_{i}^{N}\sum_{j}^{N}w_{i,j}\left(X_{i}-\overset{-}{X}\right)(X_{j}-\overset{-}{X})}{{\left(X_{i}-\overset{-}{X}\right)}^{2}}\;i,j=1,\ldots ,N=57;i\neq j$$

*Local Moran’s*

(3) $$I_{i}=\frac{X_{i}-\overset{-}{X}}{S_{i}^{2}}\sum_{j}^{N}w_{i,j}(X_{ij}-\overset{-}{X})\;S_{i}^{2}=\frac{\sum_{j}^{N}{{(X}_{j}-\overset{-}{X})}^{2}}{N-1}-{\overset{-}{X}}^{2}\;i,j\;1,\ldots ,N=57;i\neq j$$

*Getis-Ord*

(4) $$G=\frac{\sum_{i}^{N}\sum_{j}^{N}w_{i,j}X_{i}X_{j}}{\sum_{i}^{N}\sum_{j}^{N}X_{i}X_{j}}\;i,j=1,\ldots ,N=57;i\neq j$$

This study used different spatial techniques from spatial autocorrelation using global Moran’s I and Hotspot analysis using Getis-Ord Gi statistics. At the same time, analytical tools were also used in the contribution of the basic processing of a statistical dataset and its subsequent analysis, methods of thematic cartography, comparative data analysis at the level of the districts of Slovakia, spatial autocorrelation, and deduction and synthesis of acquired knowledge.

## 3. Results

**Figure 2:**
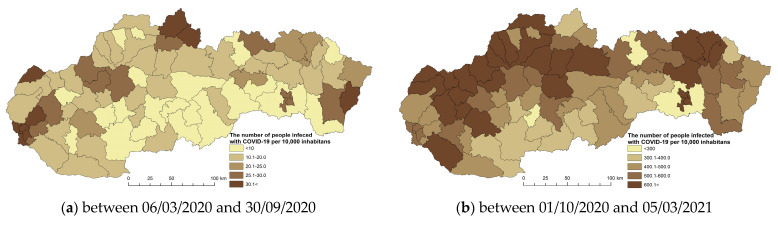

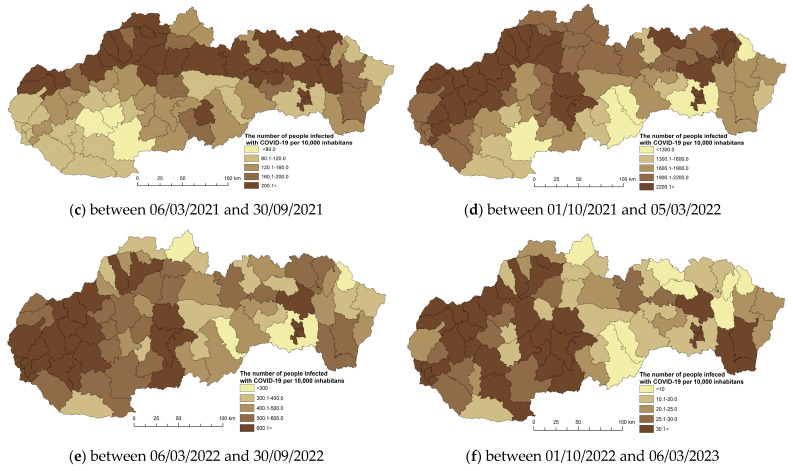
The number of people infected with COVID-19 per 10,000 inhabitants in Slovakia between 6 March 2020 and 30 September 2020, and between 1 October 2022 and 6 March 2023.

Since 2020, the world has experienced a pandemic, the consequences of which have been unprecedented in the last century. Life in the world, including in Slovakia, slowed down significantly for some time. Globalization played one of the important aspects in the spread of this disease, which, in addition to its positive effect, also has its risks. We detected the first case of COVID-19 in Slovakia on 6 March 2020. It was 4 months after its first discovery in China. The disease was imported to Slovakia from 52 countries. Most cases were from Austria, the United Kingdom, and Germany. Due to the threat to public health, the government of the Slovak Republic declared a state of emergency on the territory of Slovakia on 11 March 2020. As of 6 March 2023, since the beginning of the pandemic, 1,860,013 residents in Slovakia had been infected and registered (https://covid-19.nczisk.sk/sk) [49]. In the development of the number of people infected with the disease COVID-19 in Slovakia, we recorded alternating waves of infection with higher and lower values over three years. Officially, three waves of infection were recorded in Slovakia. The first wave (March 2020 to June 2020), the second wave (August 2020 to May 2021), and the third wave (September 2021 to May 2022). For a more detailed analysis of the infection development, we evaluated the overall situation over six periods chosen by us, which were divided into equal periods. Figure 2a–f documents the development of the infection in the observed periods from the point of view of regional differentiation in the districts of Slovakia.

The indicator of the number of infected with COVID-19/10,000 inhabitants in Slovakia shows significant spatial differences. These are related to the period in which we monitor the indicator. It is important to note the fact that the monitored values of the indicator differed significantly in the monitored periods. We recorded the lowest values of infected in the first and sixth periods. In the first period, a very important role was played by strict measures that were introduced, such as curfews, closing schools and restaurants, closing airports, traffic restrictions, and others. The first wave was handled well with few infected and casualties. By introducing hard lockdowns at the very beginning, the rapid onset of the pandemic was caught. The catastrophic scenario of the Ministry of Health’s analysts, that the healthcare system would need five times more lung ventilation than it had available, did not come true. At the same time, human solidarity helped. From the provincial point of view, the highest concentration of infected people was recorded in the districts of Bratislava, Pezinok, Námestovo, Tvrdošín, and Sobrance (Figure 2a). The number of infected residents in Slovakia gradually increased. In the second stage of monitoring, the highest values (600 infected/10,000 inhabitants) were recorded mainly in the districts of northern Slovakia. This wave had a worse impact, collapsing healthcare, and many infected and dead, despite the already available vaccination against the virus. The management of the pandemic was unmanageable, as preparation, planning, routing, and testing were neglected. During the wave, there were many chaotic political decisions that were often without a scientific basis. As an example, we present widespread testing, permitted numbers of people at cultural and other events. At the same time, these decisions were often inappropriately communicated to the public (Figure 2b). The third observed period is more favorable from the point of view of the number of infected people because the maximum number of infected people reached more than 200 infected people/10,000 population. This period is the third wave of the pandemic in Slovakia. Despite the fact that a vaccine was available, the situation was still catastrophic with a high death toll and a strain on hospitals. The politicization of the pandemic and vaccination was also a problem. Vaccination has had mixed support across the political spectrum, with many hoaxes and misinformation (Figure 2c).

The highest values of the number of infected in the period between 1 October 2021 and 5 March 2022 reached 2200.1 infected/10,000 inhabitants (Figure 2d). The highest concentration of these values was recorded in the districts of the northwestern part of Slovakia. We are following the trend of moderate decline for the next period (Figure 2e). Figure 2f documents the situation in the last period. As of 2023, the COVID-19 measures have been lifted, the vaccination campaign is minimal, and hospitals are handling the rush of patients. Despite this, the coronavirus pandemic still exists.

### 3.1. COVID-19 in Slovakia

#### Spatial Autocorrelation

**Figure 3:**
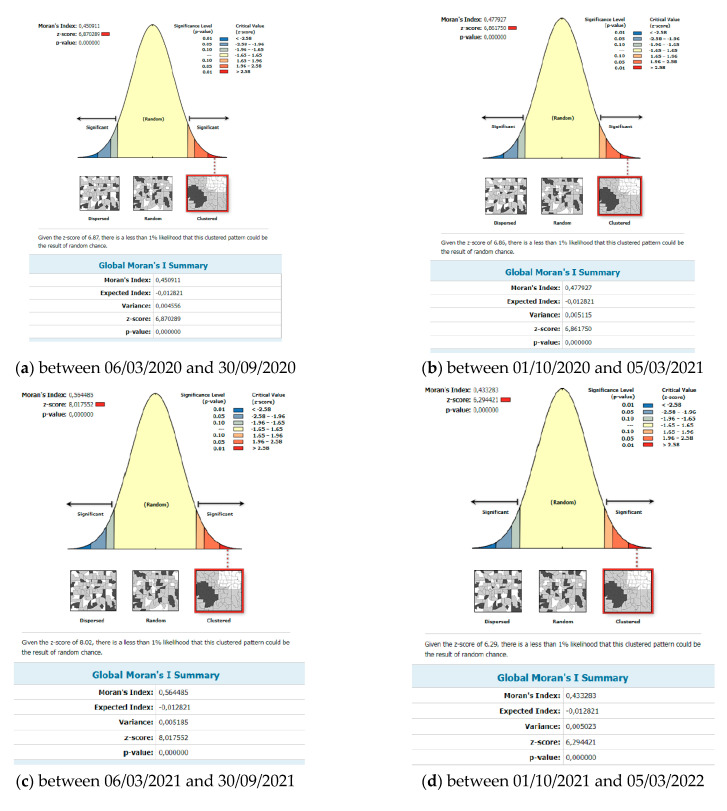

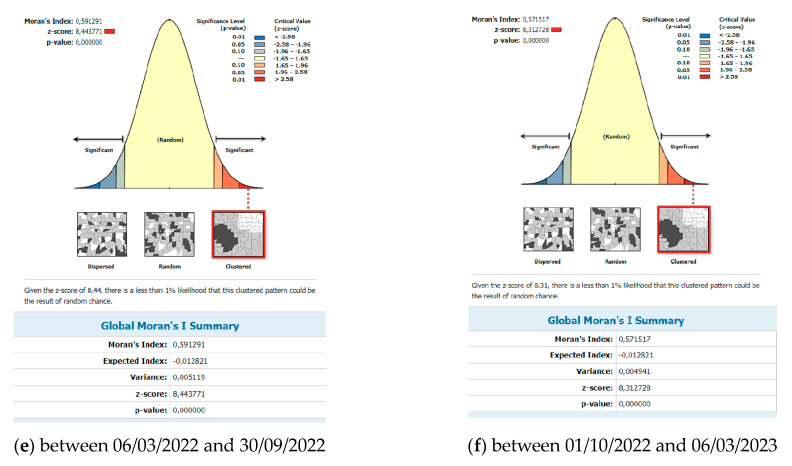
Final statistics for the number of people infected with COVID-19 per 10,000 inhabitants in Slovakia between 6 March 2020 and 30 September 2020, and between 1 October 2022 and 6 March 2023.

**Figure 4:**
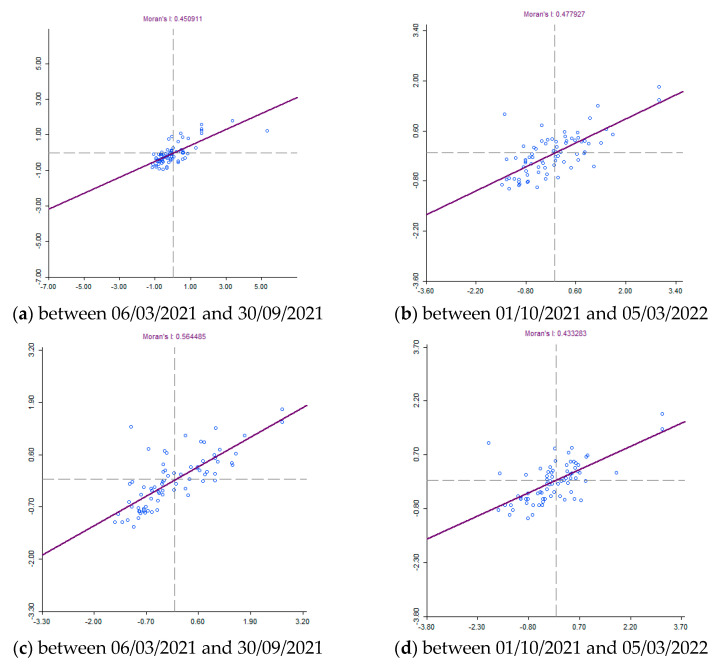

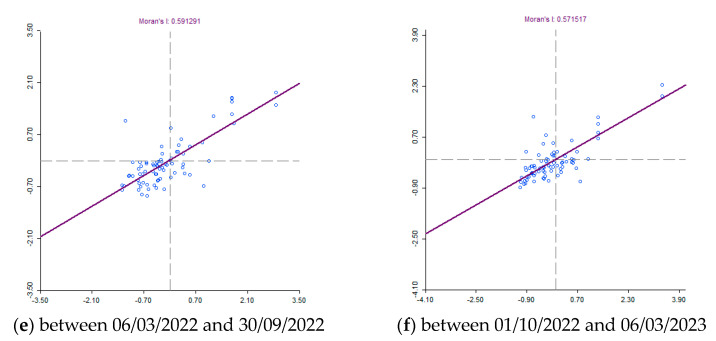
Moran’s I diagram for the number of people infected with COVID-19 per 10,000 inhabitants in Slovakia between 6 March 2020 and 30 September 2020, and between 1 October 2022 and 6 March 2023.

Knowing when and where outbreaks occur can lead to understanding the underlying causes of the COVID-19 virus and potentially predicting future outbreaks. There are various methods or techniques to reveal spatial patterns of disease, including cluster detection, hotspot analysis, and regression models. Various spatial statistical techniques for uncovering clusters are included in some geographic information system (GIS) software packages, as well as in various stand-alone programs. These programs include, for example, GeoDa. Global spatial cluster analysis and spatial correlations of the epidemic of COVID-19 between the districts of Slovakia were defined according to Global Moran I calculations, the values of which were higher than 0 (positive) for COVID-19 for all monitored periods (Figure 3). Moran’s indices took on values from 0.433283 to 0.591291. This is documented in Figure 4. The Z-score and Moran’s I indicate that the incidence of COVID-19 was positively and spatially correlated between regions. Moran’s I results show that positivity for COVID-19 was spatially clustered in the study area.

Local spatial cluster analysis: The results of Local Moran′s I analysis revealed statistically significant locations. Figure 4 documents the results as a geographical distribution of occurrence. In general, the Local Moran I maps indicated that most counties exhibited high–high or low–low clustering types. There are several outliers that show low–high and high–low local spatial autocorrelation over the entire time span of the study. We present a more detailed analysis of the results below.

In an attempt to investigate and explain the spatial patterns of the COVID-19 infection in Slovakia, we used spatial autocorrelation techniques. These were specific techniques—global and local. The results showed that there is a relatively large geographical difference in significant clusters of the incidence rate of COVID-19 infection between the districts of Slovakia. This study demonstrated the great importance of research on how a public health emergency can affect the lives of the population. Our contribution should be a starting point for research in the given issue. Health–geographical approaches will continue to play a critical role even after this pandemic is over. At the same time, it is important to realize the dynamics of the disease’s development and emerging new mutations. Like many phenomena, the COVID-19 pandemic has shown us its geography. In this paper, we analyzed the cumulative number of confirmed cases of COVID-19/10,000 inhabitants as a variable in the districts of Slovakia. A spatial weight matrix was selected on the basis of geographical proximity, global Moran’s I index, *p*-value, and z-score.

**Figure 5:**
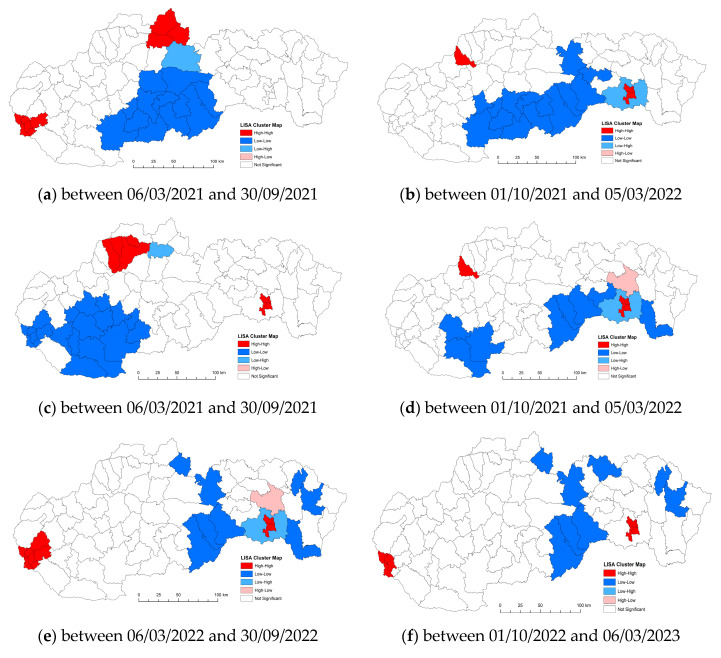
Regionalization of Slovakia based on the LISA analysis for the number of people infected with COVID-19 per 10,000 inhabitants in Slovakia between 6 March 2020 and 30 September 2020, and between 1 October 2022 and 6 March 2023.

The number of confirmed cases of COVID-19/10,000 population was calculated using GeoDa software to clarify global spatial correlation characteristics. The use of spatial reference data on the number of people infected with the disease COVID-19 in Slovakia offers us an understanding of spatial-analytical links in this area of interest. With the spatial autocorrelation method, clusters of regions can be defined from the point of view of the monitored indicator. On the territory of Slovakia in the first period, we identified one compact region in the low–low quadrant, which is made up of 12 districts in the southern part of central Slovakia. Isolated in the next quadrant (high–high) are the districts of Námestovo, Tvrdošín, and Dolný Kubín. In the western part of Slovakia, they are joined by the districts of Senec and Bratislava I–V. By analyzing the monitored indicator in this period, we identified 14 districts, mainly in the southern part of Slovakia, which we classified in the low–low quadrant. The manifestation of positive spatial autocorrelation is also observed in the districts of Ilava and Košice I–IV, which belonged to the high–high quadrant. The negative spatial autocorrelation is in the Košice-okolie district and belongs to the low-high quadrant. In the next monitored period between 6 March 2021 and 30 September 2021, the situation from the point of view of the monitored indicator changed, primarily in the localization of the created clusters. In the western part of Slovakia, a rather significant low–low cluster was formed, which included 20 districts of Slovakia. Positive spatial autocorrelation was observed in the districts of Žilina, Kysucké Nové Mesto, Bytča, Považská Bystrica, and Košice I–IV. The given group of districts was included in the high–high quadrant. Changes were observed in analyses of the phenomenon of spatial autocorrelation in the given period. We can conclude that on the basis of the spatial autocorrelation created in this period, the districts of Ilava and Košice I–IV were located in the high–high quadrant. Isolated in the next quadrant (low–low) are three districts in the western part (Nitra, Levice, Nové Zámky). Towards the east, they are joined by the districts of Rimavská Sobota, Revúca, Rožňava, Gelnica, and Trebišov. Prešov district appears separately in the high–low quadrant and the Košice-okolie district in the low–high quadrant. In the period between 6 March 2022 and 30 September 2022, we observed manifestations of positive but also negative spatial autocorrelation. The first cluster of the high–high type in this period is located in the districts Košice I–IV, Bratislava I–V, Pezinok, and Senec. The localization of the districts that were included in the low–low quadrant is represented in a mosaic. These were eight districts, mainly in the eastern part of Slovakia. High–high cluster with districts Bratislava I–V and Košice I–IV. Districts located in several parts of Slovakia (Tvdošín, Poprad, Stará Ľubovňa, Stropkov, Humenné, Rožňava, Revúca, Rimavská Sobota) are characterized by positive spatial autocorrelation, specifically by representation in the low–low quadrant (Figure 5a–f).

## 4. Discussion

The aim of this study was to reveal the spatial patterns of cases of COVID-19 at the level of the districts of Slovakia. Our primary goal was to identify clusters spatially. According to our knowledge, this is the first ever study carried out in Slovakia. Influenza pandemics were and remain part of the life of the population. However, when, where, and in what strength it will reappear cannot be seriously predicted on the basis of the current knowledge. The viral outbreak of COVID-19, which is declared a global pandemic, has overwhelmed the healthcare system with an increasing number of patients. The rising rate of infection presented its own challenges, which burdened the health sector as well as the global economy [49]. It is quite likely that COVID-19 was already here before the first case was registered on 3 June 2020. The problem arises when identifying because the so-called severe, protracted infections were considered influenza. Looking back shows that many cases showed all the typical signs of coronavirus infection, but the tests were not done then [50]. The genetic pandemic of COVID-19 is a powerful reminder that urbanization has changed the way people live and work. Therefore, there is a need to strengthen systems and local capacities to prevent the spread of infectious diseases [51]. According to Suligowski and Ciupa [52], several waves of COVID-19 caused by different variants of SARS-CoV-2 have been recorded around the world. During this period, many publications have been published describing the influence of various factors such as environmental, social, and economic factors on the spread of COVID-19. The COVID-19 pandemic has seriously affected European countries and their healthcare systems. Country risk assessment is an integral part of pandemic preparedness and response. The key EU body that assesses the risk of virus spread and the impact on public health is the European Center for Disease Prevention and Control. One of the ways to prevent these risks is to identify risk regions. Spatial autocorrelation also offers this possibility. Many health services in severely affected countries are undergoing decentralization and fragmentation [53]. The COVID-19 virus has caused widespread health problems in all countries. The database of Covid19 diseases in the world and in Slovakia was available from sources [54,55]. The findings showed that the spatial distribution of COVID-19 in Slovakia is heterogeneous, and cases were concentrated in all regions depending on the period in which they were monitored. In addition, our research shows that geographic and temporal analyses will be very important in the future. Similarly, surveillance-based disease data will benefit the management of viral infections such as COVID-19. In addition, this strategy takes into account the determination of the so-called different zones throughout the country. Likely, COVID-19 was here before we first noticed it, except we thought of those severe, protracted infections as influenza/flu. However, in retrospect, many cases showed all the typical signs of coronavirus infection, but the tests were not done at the time [51]. 

According to the global Moran’s I values, we can conclude that there is a spatial pattern throughout the country. The values remained stable at the beginning, but in subsequent periods, the cases increased and decreased again with the blockade. Local spatial autocorrelation analysis makes it possible to find outliers and local correlations in Slovakia. Low–low and high–high clusters were dominant throughout the weeks, with a few outliers. The local correlation characteristics varied over time and space, which was stable for several weeks at the beginning of the time period. Low–low and high–high clusters moved across the western and eastern parts of the country. During the monitored period, an increase was observed throughout the country due to the activation of intercity mobility and the relaxation of quarantine measures. This situation actually reveals the importance of the applicability and traceability of the measures rather than their strictness. The introduced measures were adapted to individual countries and to the general personal characteristics of the country’s inhabitants. Measures should not detract from their main purpose and, most importantly, measures should be traceable.

The Moran’s I measurements also directly overlap with the development in the timeline of the COVID-19 pandemic in Slovakia and provide important information for the spatial analysis of the pandemic. Moreover, the first-degree neighbors were statistically significant. It was found that national measures, rather than regional measures, significantly reduced the rate of spread in certain periods. As a result, city-level policies may not produce the expected results. The COVID-19 pandemic, the spatio-temporal effects of which were discussed in this study in Slovakia, affected the population in various areas. Spatial assessment of a pandemic that affects sustainable development goals and hinders sustainability, especially health and economy in countries, can influence decision makers. Thus, the problem can turn into an easy-to-solve local problem instead of a difficult-to-solve national problem.

## 5. Conclusions

Using spatial methods for modeling the occurrence of COVID-19 in Slovakia is important for improving current strategies and predicting the future. We found several high-risk clusters in different regions of Slovakia, and the socioeconomic conditions of the affected districts could be important factors for the grouping of cases. In the future, the findings of our study could enable a narrower focus on the incidence of COVID-19 and socioeconomic predictors to mitigate the risk of the disease and control it in risk regions. The rate of spread of the COVID-19 epidemic in Slovakia has obvious spatial differences. Population distribution, transport accessibility, average temperature, and conditions of medical facilities had a significant impact on the rate of spread of the epidemic. The analyses confirm the significant spatial aspect of the distribution of the monitored indicator, which is also manifested at the regional level. This fact is also reflected in the specification of significant differences in several areas or from the social, economic, and economic aspects, especially in the area of east and west. Much research on COVID-19 still needs to be done in Slovakia to better understand the dynamics of the pandemic, appropriateness, and adaptation to the national context.

## Data Availability

The data presented in this study are available on request from the corresponding author. The data are not publicly available due to privacy reasons.
